# Biodistribution and Toxicity of Micellar Platinum Nanoparticles in Mice via Intravenous Administration

**DOI:** 10.3390/nano8060410

**Published:** 2018-06-07

**Authors:** Anna L. Brown, Marc P. Kai, Allison N. DuRoss, Gaurav Sahay, Conroy Sun

**Affiliations:** 1 Department of Pharmaceutical Sciences, College of Pharmacy, Oregon State University, 2730 SW Moody Ave, Portland, OR 97201, USA; broanna@ohsu.edu (A.L.B.); marc.p.kai@gmail.com (M.P.K.); duross@ohsu.edu (A.N.D.); sahay@ohsu.edu (G.S.); 2 Department of Biomedical Engineering, School of Medicine, Oregon Health & Science University, 2730 SW Moody Ave, Portland, OR 97201, USA; 3 Department of Radiation Medicine, School of Medicine, Oregon Health & Science University, 3181 S.W. Sam Jackson Park Road, Portland, OR 97239, USA

**Keywords:** platinum, nanoparticle, noble metal nanoparticles, in vivo toxicity, bioaccumulation

## Abstract

Platinum nanoparticles (PtNPs) have shown promise as diagnostic and therapeutic agents due to their unique physiochemical properties. However, critical parameters, such as toxicity and accumulation at both desired and other tissues, remain a significant concern in the clinical translation of these nanomaterials. Here, we examine the cytotoxicity, biodistribution, and effect on clearance organ function of an intravenously administered polyethylene glycol (PEG) -ylated PtNP construct. We synthesized hydrophobic PtNPs and assembled them into aqueous micelles with the lipid-polymer conjugate 1,2-distearoyl-sn-glycero-3-phosphoethanolamine (DSPE)-PEG (PtNP: DSPE-PEG, ~70 nm). This construct was well tolerated in mice receiving up to 15 mg platinum per kg body weight with no observed loss in weight, plasma chemistry within normal healthy ranges, and normal histopathology of organs after three weeks. Platinum quantification studies (inductively-coupled plasma mass spectroscopy (ICP-MS)) were also performed to assess biodistribution of PtNPs. The findings of this study are consistent with the in vivo accumulation of metal nanomaterials and further highlight the need to address clearance when designing nanomaterials for medical applications.

## 1. Introduction

Noble metal nanoparticles are an emerging class of materials that may soon significantly impact human health through their widespread use and potential as functional biomaterials [1,2,3]. These nanoscale engineered particles possess unique electronic, physical, and chemical properties that are being exploited in biomedical applications, such as diagnostic assays [4], molecular imaging [5], implants [6], and drug delivery strategies [7]. Gold-based nanomaterials have been widely investigated in nanomedicine due to their ease of synthesis and the inert chemical nature of this element. Silver nanoparticles (AgNPs) are commonly used anti-microbial agents, and have predictably been shown to be more toxic in biological models relative to less chemically reactive gold nanoparticles (AuNPs) [8]. Recently, platinum nanoparticles (PtNP) have gained a great deal of attention in nanomedicine [9]. The intrinsic photophysical properties of these elemental particles have been explored in novel applications, such as photothermal therapy [10], radiation dose enhancement [11], and computed tomography (CT) X-ray contrast [12]. In addition, increased interest has been focused on the catalytic properties of PtNPs in a biological context as synthetic nanoenzymes for selective radical scavenging in the treatment of oxidative stress diseases [9,13,14,15,16].

A major hurdle to the clinical translation of these nanomedicines is the uncertain toxicity of these particles. Nanomaterial size and surface chemistry are fundamental characteristics that affect both the pharmacokinetics and biological response to these particles. In addition, it has been well established that the physiochemical properties of the material dictate tissue and cellular uptake, transportation, protein interaction, toxicity and ionization of the metal cores. Toxicity studies of noble metal nanomaterials, most notably AuNPs, have returned mixed results with difficulty in establishing strong relationships due to the wide variety of materials and experimental conditions reported [17]. However, some general conclusions on key parameters affecting their biodistribution and toxicity are beginning to be distilled from this body of work [17,18]. Unfortunately, less is known about PtNP toxicity beyond in vitro studies. Elemental platinum (Pt) is less chemically reactive than gold and has been shown to have intrinsic biological stability and tolerability [19]. In its zero oxidation state, Pt has been be shown to have no clinical consequence on women receiving breast implants containing the metal at ppm levels [6]. Increasing development of applications requiring administration by parenteral routes and larger quantities of Pt warrant more thorough studies of PtNPs.

Herein, we report the in vivo biodistribution and toxicity of a PtNP construct comprised of 2–4 nm Pt cores encapsulated in a polymer-lipid micelle (1,2-distearoyl-sn-glycero-3-phosphoethanolamine-*N*-[(polyethylene glycol)-2000] (DSPE-PEG_2000_)) with overall diameter of ~70 nm. The hydrophobic PtNP cores were synthesized by a method adapted from Zhang et al., utilizing oleylamine as a surfactant [20]. To enable the use of these nanoparticles in biological systems, we developed an encapsulation process to form uniform hydrophilic clusters of PtNPs. In this construct, the PEGylated surface serves to enhance biological compatibility, mitigate opsonization, and increase circulation time. By aggregating these nanocrystals into larger clusters, we avoid rapid renal clearance while maintaining a potential route of elimination upon subsequent degradation of the carrier [21,22]. In addition, the ubiquitous use of PEG and their functional derivatives to control surface chemistries, extend circulations times, and enable targeting strategies, make this platform a relevant model for these studies. We expect this work, demonstrating the relative biocompatibility of PtNPs, will contribute to the growing literature documenting the safety and response to nanomaterial administration. In particular, these results highlight the need to thoroughly examine the biodistribution and fate of noble metal nanoparticles in vivo.

## 2. Materials and Methods

### 2.1. Materials

All materials were obtained from commercial suppliers and used without further processing or purification. Platinum(II) acetylacetonate (Pt(acac)_2_), oleylamine, ethanol, cyclohexane, nitric acid, hydrochloric acid, tin(II) chloride, tetrahydrofuran (THF), and dimethyl sulfoxide (DMSO) were obtained from Fisher Scientific (Waltham, MA, USA). 1,2-distearoyl-sn-glycero-3-phosphoethanolamine-*N*-[(polyethylene glycol)-2000] (DSPE-PEG_2000_) was purchased from Avanti Polar Lipids (Alabaster, AL, USA). Ultra-15 10,000 molecular weight cutoff (MWCO) centrifugal filter concentrators and 0.8-µm syringe filters were obtained from VWR (Radnor, PA, USA) RPMI 1640, Dulbecco’s Modified Eagle Medium, fetal-bovine serum, penicillin-streptomycin, alamarBlue, Blue-Green Live/Dead Assay, 10% formalin, trypsin-EDTA, and Dulbecco’s phosphate-buffered saline (PBS) were purchased from Life Technologies (Carlsbad, CA, USA).

### 2.2. Platinum Nanoparticle Synthesis

PtNPs were synthesized by reduction of platinum(II) acetylacetonate (50 mM, 10 mL) in oleylamine by addition of an equal volume borane tert-butylamine (0.92 M, 10 mL) at 100 °C under an inert atmosphere. Once the solution turned black (~30 s), particles were allowed to ripen for 20 min at 100 °C. The suspension was cooled to room temperature, and PtNPs were precipitated in 200 mL ethanol followed by collection via centrifugation (20 min, ca. 5000× *g*). Particles were then suspended in 10 mL cyclohexane and briefly sonicated to disperse hydrophobic contaminants, washed in 50 mL ethanol, and recollected by centrifugation. PtNP cores were stored in ethanol until further use.

### 2.3. Micelle Assembly

PtNPs were dried under nitrogen, quantified by dry weight, and re-suspended in THF at 1–10 mg/mL. DSPE-PEG was dissolved in THF and mixed with PtNPs to achieve exact weight and concentration ratios. 500 μL of these suspensions were slowly injected into 10 mL of 18 MΩ water, with rapid stirring. Micelles were allowed to stir for 2 h, and then dialyzed against 18 MΩ water using 10 kMWCO regenerated cellulose dialysis tubing (Snake Skin, Thermo Fisher Scientific) overnight. For larger scale synthesis of micelles for in vivo studies PtNPs were suspended in 15 mL THF at 2 mg/mL concentration, and added to 300 mg dried DSPE-PEG in a glass scintillation vial and re-suspended by emersion by sonication (20 min). After cooling to r.t., micelles were assembled by injecting the THF suspension though a 100 μL micropipette tip into 150 mL 18 MΩ water, stirred for 1 h, and dialyzed overnight twice against 4 L 18 MΩ water to removed residual THF. Assembled micelles were concentrated using an Amicon Ultra-15 10,000 MWCO centrifugal filter concentrator for 10 min at ca. 5000× *g*. Micelles were passed through a 0.8-μm syringe prior to Pt quantification and biological assays.

### 2.4. Characterization

Size distribution of the particle suspension was evaluated using dynamic light scattering (DLS, Malvern Zetasizer NanoDS, Westborough, MA, USA). Transmission electron microscopy (TEM) was performed on a Tecnai F-20 TEM (FEI, Hillsboro, OR, USA) operating at 200 kV. Samples were prepared by drop casting onto a carbon coated copper grid (Ted Pella, Redding, CA, USA), and allowed to air dry. XRD data were collected in focused beam (Bragg−Brentano) geometry on a Rigaku Ultima IV X-ray diffraction system (Woodlands, TX, USA) using graphite monochromatized Cu Kα radiation. Scans were performed over the angular range 20−70° 2θ at a scan rate of 0.25°/min at r.t.

### 2.5. Platinum Quantification

A 100 μL aliquot of PtNPs was digested in an equal amount of aqua regia in polypropylene test tubes for 12 h at room temperature. After the digestion, 200 μL of a freshly-prepared 4 M–0.4 M HCl-SnCl_2_ solution was added, along with an additional 200 μL of water. The absorbance of the resulting Pt-SnCl_2_ complex solution was measured on a Tecan Infinite^®^ M200 Pro plate reader (Mannedorf, Switzerland) at 403 nm against a standard curve to yield a Pt concentration.

### 2.6. In Vitro Cytotoxicity

Cells were seeded in 100 μL of media (RMPI 1640 Medium for 4T1-luc-tdTomato and HepG2; DMEM for NIH3T3; all media was supplemented with 10% fetal bovine serum and 1% penicillin-streptomycin) at a density of 1000 cells into a 96-well microtiter plate. Cells were allowed to adhere for 12 h and subsequently incubated with either PtNPs or DMSO at concentrations ranging from 0.0512 to 2560 µM Pt, 10% for DMSO, for 1 or 4 h at 37 °C in a humidified 5% CO_2_ atmosphere. After the incubation period, all media/particles were aspirated off cells. 100 μL fresh medium was added and cells were allowed to proliferate for 72 h, followed by the addition of 10 μL alamarBlue^®^ assay reagent (Thermo Fisher Scientific). Plates then incubated at r.t. for 24 h to stabilize the fluorescent signal. The viability of the cells was expressed as a percentage of the viability of cells grown in the absence of PtNP or DMSO. In addition, the cytotoxicity of the PtNP micelle complex was assessed by fluorescence-based LIVE/DEAD assay (Thermo Fisher Scientific).

### 2.7. Animals

All experiments involving mice were performed in accordance with the National Research Council’s Guide to Care and Use of Laboratory Animals (1996), under an animal use protocol approved by the Oregon Health and Sciences University (OHSU) Institutional Animal Care and Use Committee (IACUC) Protocol IP00000023 (approved 04/20/2015) . All studies used female BALB/c mice (6 weeks, 17–27 g, Jackson Laboratory). For the maximum tolerated dose study, naïve animals were used. For the biodistribution study, both a naïve and an orthotopic breast (4T1-luc-tdTomato) model were used. Cell cultures were prepared and maintained per vendor specifications. For the tumor-bearing mice, a 100-µL suspension of luciferase- and tdTomato-expressing 4T1 cells (1 × 10^5^ cells per mouse in PBS) was implanted into the mammary fat pad. Tumor growth was monitored by bioluminescence and fluorescence imaging (Caliper Life Sciences IVIS XRMS, Waltham, MA, USA). The biodistribution study commenced five days after tumor inoculation, once tumors were palpable.

### 2.8. Dose Escalation Study

Mice were dosed intravenously (*n* = 3) via tail-vein with saline or 5, 10, 15, or 20 mg/kg PtNPs (based on Pt content). Animals were monitored and weighed three times per week, with acceptable body weight loss specified as ≤20%, per protocol guidelines. Three weeks after injections animals were sacrificed; plasma was collected for renal and liver marker panels (Oregon State University Veterinary Diagnostic Laboratory), and organs were harvested for either inductively-coupled plasma mass spectroscopy (ICP-MS) analysis (Agilent Technologies, Santa Clara, CA, USA) (liver, spleen, lung, kidney) or histology (liver, spleen, heart, kidney) by the OHSU Histopathology Core.

### 2.9. Biodistribution

PtNPs were dosed intravenously into either naïve or tumor-bearing mice (*n* = 3) via tail-vein at 10 mg Pt/kg. An additional tumor-bearing mouse received a saline injection as a control. Twenty-four hours post-injection, blood was collected in heparin by cardiac puncture and centrifuged (300× *g*, 5 min, 4 °C) to isolate plasma from the cell fraction. The liver, spleen, lungs, heart, kidneys, and tumor (where applicable) were harvested and weighed, and either fixed in 10% formalin for histology (liver, kidney) or set aside for ICP-MS analysis (liver, spleen, lung, kidney, tumor, heart).

### 2.10. ICP-MS Analysis

Tissue sample preparation was performed by digesting whole organs (kidney, lung) or a section (liver, spleen) in aqua regia for 24 h. Deionized water was added to bring the sample to volume and an appropriate acid concentration, and the samples were stored at 4 °C until Pt analysis was completed. Inductively-coupled plasma mass spectroscopy (ICP-MS) analysis was performed and validated by the OHSU Elemental Analysis Core.

### 2.11. Data Analysis

Statistical differences between treatment groups were assessed by ANOVA and Student’s *t*-test for unpaired data between two groups. The level of significance was set at *p <* 0.05. If *p* < 0.05, Tukey-Kramer multiple comparisons post-tests was performed.

## 3. Results

**Figure 1:**
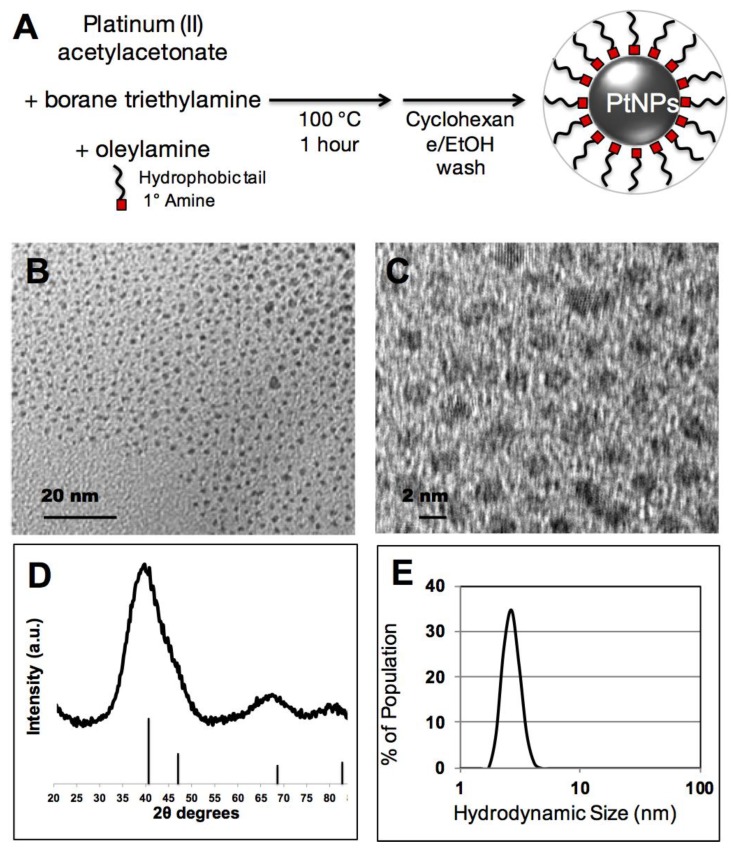
(**A**) Schematic illustration of platinum nanoparticles (PtNPs) core synthesis; (**B**) Transmission electron microscopy (TEM) of PtNP cores synthesized in oleylamine; and (**C**) high-resolution TEM of the crystalline PtNPs; (**D**) Powder X-ray diffraction confirms presence of small nanoparticles composed of crystalline platinum; (**E**) Hydrodynamic size of PtNP cores by dynamic light scattering.

In this study, we adapted a PtNP synthesis that was previously developed for noble metal nanoparticle surface catalysis and modified it for potential use in biological applications. This method exploits the solvent/surfactant properties of oleylamine and a borohydride reducing agent to convert soluble platinum(II) into an elemental platinum(0) nanocrystal (Figure 1A). As synthesized PtNP crystalline cores were 2–4 nm by TEM imaging (Figure 1B), and show crystalline diffraction spacing consistent with elemental Pt (Figure 1C). X-ray diffraction spectroscopy of dried PtNPs additionally confirms a crystalline Pt phase (Figure 1D, JPCDS #04-0802). Substantial line broadening of peaks and overlap, particularly with respect to the reflections at 40.3° and 46.8°, has been previously reported with small PtNPs [20]. In addition, the hydrodynamic size of the PtNPs in cyclohexane was measured at 2.5 nm, consistent with a 2–4 nm Pt inorganic core stabilized by an oleylamine surfactant (Figure 1E).

**Figure 2:**
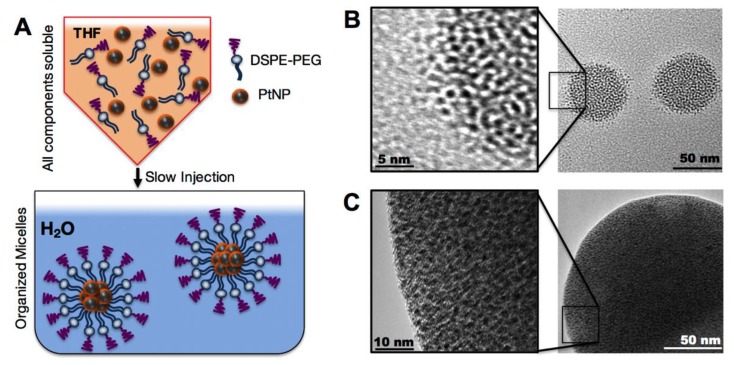
(**A**) Schematic of PtNP encapsulation in 1,2-distearoyl-sn-glycero-3-phosphoethanolamine-*N*-[(polyethylene glycol)] (DSPE-PEG) micelles; (**B**) Representative TEM of assembled micelles with peak distributions at ~70 nm; and (**C**) ~150 nm diameter.

We then assembled these hydrophobic PtNPs into micelles using the lipid-polymer conjugate (DSPE-PEG), a highly biocompatible natural lipid covalently linked to a neutral hydrophilic polymer. DSPE-PEG self-assembled in aqueous media into a micellar structure encapsulating the hydrophobic PtNPs. This construct is a PEGylated nanoparticle which should lead to a reduction in macrophage uptake and subsequent prolonged circulation times [23,24]. To assemble the PtNP: DSPE-PEG micelles, both the hydrophobic PtNPs and DSPE-PEG were dissolved in THF and then slowly injected by micropipette into rapidly stirring 18 MΩ water, enabling micelle self-assembly (Figure 2A). Variations of assembly conditions were surveyed, including PtNPs concentration in the THF phase (0.001–5 mg/mL), and PtNP to DSPE-PEG ratios by weight (0.1:1 to 1:100). The resulting PtNP encapsulated micelles ranged in mean hydrodynamic size from 50–150 nm (Supplementary Materials Figure S1). We were able to maintain distinct control over the micellar size and polydispersity by altering synthetic conditions. Assembly conditions were screened in order to determine which factors contributed to the final assembled micelle size. Transmission electron microscopy of the variations of micelles further confirm the aggregation of PtNP cores by DSPE-PEG. Figure 2B including a high ratio of DSPE-PEG to PtNPs (~70 nm mean diameter), while a low ratio of DSPE-PEG to PtNPs (Figure 2C) results in a larger (~150 nm mean diameter) assembly. These images also illustrate that this ratio imparts different levels of stability on the final micelle construct.

Given the high degree of control over the micelle assembly size, we proceeded to biocompatibility and biodistribution studies with micelles that were less than 100 nm by DLS, which had an excess of DSPE-PEG coating imparting a stealth property via a decrease in serum protein interactions. We assembled PtNP:DSPE-PEG micelles with a weight ratio of 1:10, and 2 mg Pt/mL. These micelles had a hydrodynamic radius of ~70 nm with a polydispersity index of 0.06. A large batch of micelles were assembled using the method described above, dialyzed twice against 18 MΩ water to remove excess THF, and concentrated using a centrifugal filter unit, and finally passed through a 0.8 μm syringe filter for sterility. This preparation was then quantified for Pt concentration using a tin chloride complex assay [25]. By digesting samples in aqua regia we were able to adapt this method for use with the above PtNPs, despite their composition of crystalline platinum(0). Here a typical concentration of Pt in the assembled and purified micelle solutions was 2.5 mg/mL. Higher concentration of the PtNP:DSPE-PEG resulted in a viscous solution making this a limiting factor for these biological studies.

**Figure 3:**
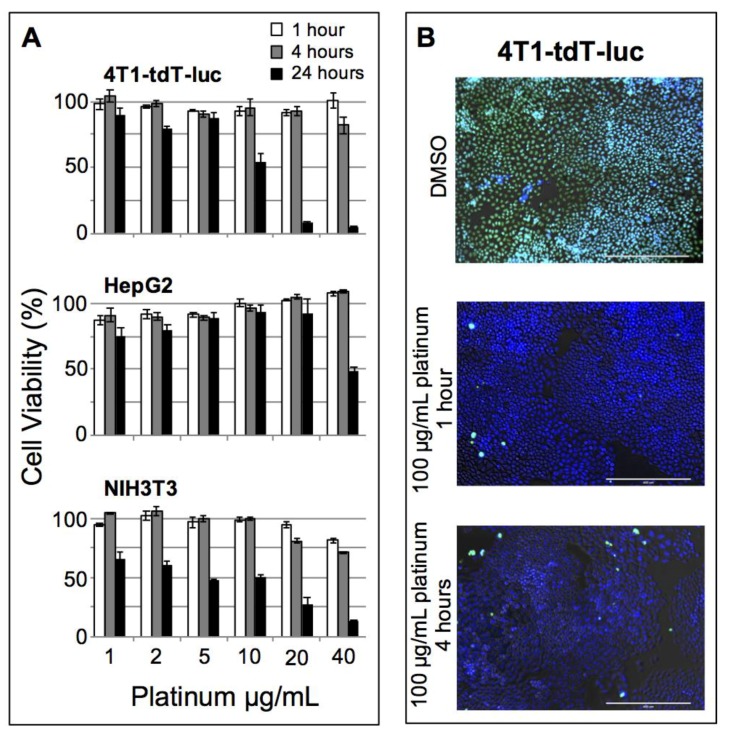
(**A**) Cell viability as determined by alamarBlue assay for 4T1-tdT-luc, HepG2, and NIH3T3 cells incubated with PtNPs DSPE-PEG micelles for 1, 4, and 24 h; (**B**) Live/Dead (Blue–live, Green–dead) fluorescence viability assay of 4T1 cells incubated with micelles at 4 h.

Cytotoxicity was examined in three cell lines of interest for cancer research, 4T1-tdTomato-luc (murine breast cancer), HepG2 (human hepatocellular carcinoma), and NIH/3T3 (murine fibroblast), using the fluorescent alamarBlue cell viability assay. Cells were plated at 1000 cells/well and dosed with particles at concentrations ranging from 1.0 to 40 μg Pt/mL, or with DMSO as a positive control (Figure 3A). No significant nanoparticle induced toxicity was observed in any of the cell lines tested up to 10 μg/mL after a 1- or 4-h incubation. A live/dead fluorescence kit was also used to visually confirm cell viability at these time points for 4T1-tdTomato-luc (Figure 3B). DMSO-treated cells (positive control) appeared green (dead), whereas cells treated with PtNP:DSPE-PEG micelles were blue (live) and exhibited insignificant cell death relative to untreated cells. The onset of toxicity was observed after 24 h exposure in both murine cell lines, 4T1-tdTomato-luc and NIH/3T3, while no significant drop in viability was found until reaching the highest concentration (40 μg/mL). Establishing the baseline safety and biocompatibility of this nanomaterial in the absence of additional drug or applied radiation provides the framework for subsequent functional development of these nanoparticles.

**Figure 4:**
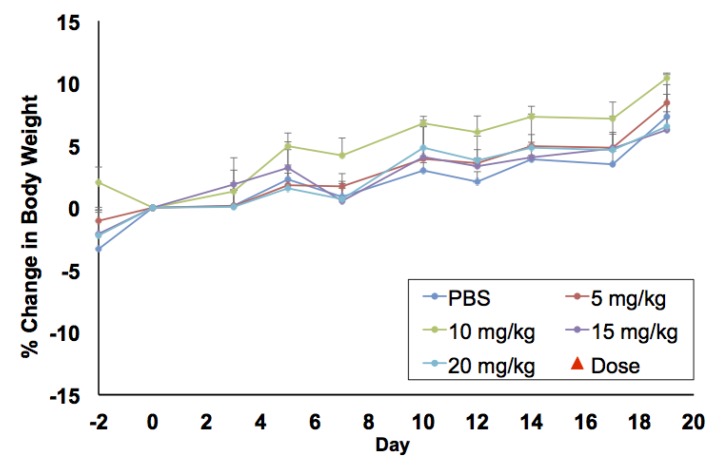
Weight change in mice injected with increasing doses of PtNP:DSPE-PEG micelles or phosphate buffered saline (control). *N* = 3 per treatment group.

**Figure 5:**
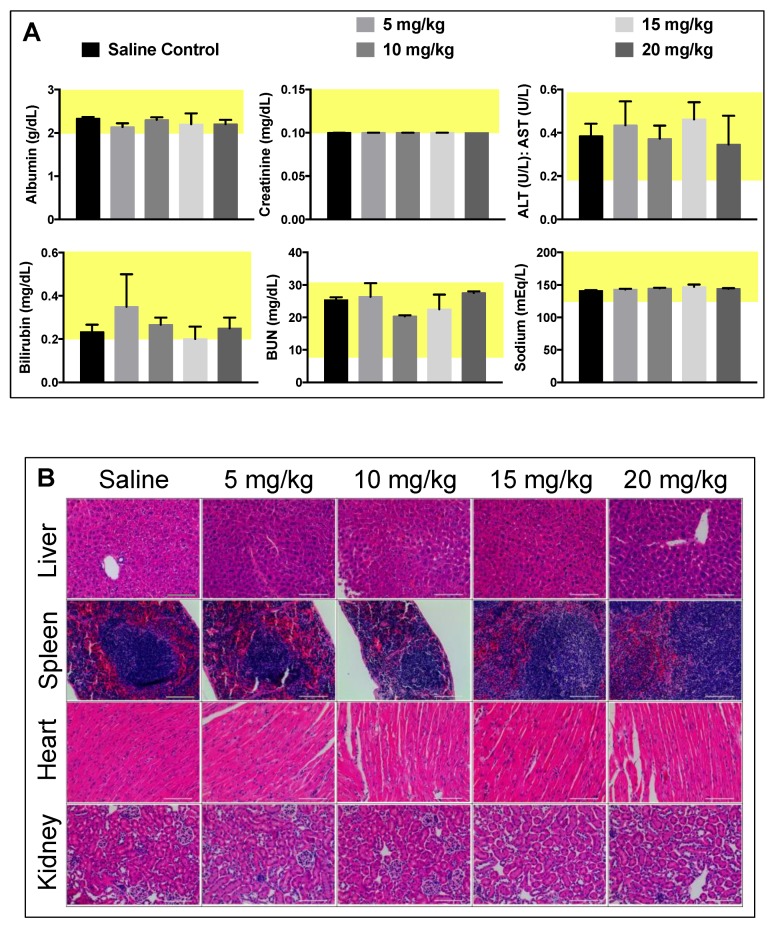
Plasma toxicity and histopathology show no toxicity effects from PtNP:DSPE-PEG micelles up to 15 mg/kg of platinum. (**A**) Serum levels of common toxicity markers from plasma collected at termination of the tolerated dose study (3 weeks); (**B**) H & E histopathology of select organs collected at termination of the study.

In vitro biocompatibility studies were complemented by in vivo maximum tolerated dose (MTD) and biodistribution studies in BALB/c mice. For MTD experiments, a single dose of PtNP:DSPE-PEG 5, 10, 15, and 20 mg Pt/kg body weight, or a saline control, was administered intravenously to healthy female mice (*n* = 3/group). Animals were weighed three times a week to monitor any weight changes that could indicate toxicity (Figure 4). No loss in body weight was observed and no adverse physical or behavioral effects were noted relative to control animals. This suggests minimal nanoparticle induced toxicity up a dose of 20 mg/kg. At the termination of the study, blood was collected, and plasma was analyzed for several clinical markers of organ health. Hepatic toxicity was assessed by alanine transaminase (ALT), aspartate transaminase (AST), bilirubin, albumin, and blood urea nitrogen (BUN) levels. Renal toxicity was measured by creatinine, sodium, albumin, and BUN levels. Results from this analysis showed no abnormal levels compared to the saline control group (Figure 5A). Furthermore, all levels were found to be within normal range for female BALB/c mice, denoted by the highlighted (yellow) region of each graph [26].

At the termination of the MTD study, organs were harvested, fixed, and sectioned for histopathology to assess PtNP:DSPE-PEG micelle biocompatibility and impact on cellular structures. No noticeable inflammation or cell recruitment was observed by hematoxylin and eosin (H&E) staining in mice injected with particles compared to the saline controls (Figure 5B). The liver, spleen, and kidneys were assessed as clearance organs, which were likely to have structural cellular response to high levels of injected nanoparticles. The heart was also assayed to evaluate potential cardiotoxicity. Upon analysis and comparison to control tissues, organ structure and morphology was unaffected in all organs tested by H&E staining. Only spleen samples of mice that received the highest dose (20 mg/kg) showed slight changes architecture with decreased white pulp. Further quantitative analysis of additional tissue slices is necessary to investigate the potential splenic toxicological impact.

**Figure 6:**
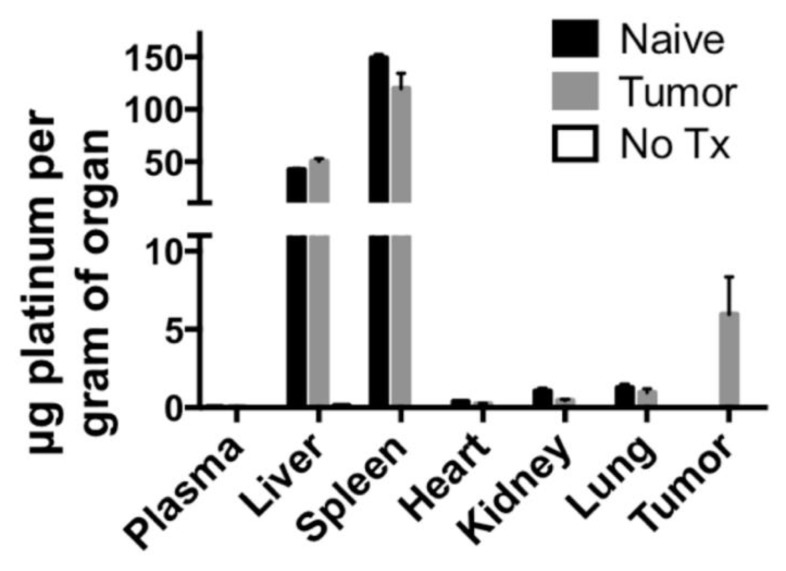
Biodistribution of PtNP:DSPE-PEG micelles in BALB/c mice (naïve, black), BALB/c mice bearing 4T1 tumors (tumor, gray), and BALB/c mice receiving saline (No Tx, white). Micelles were injected at 10 mg Pt/kg body weight, and tissues were harvested 24 h post-injection.

Short-term biodistribution studies were performed by injection of PtNP:DSPE-PEG micelles at a concentration of 10 mg Pt/kg body weight in non-tumor bearing mice, 4T1 tumor bearing syngeneic mice or a saline control. After 24 h animals were sacrificed, and organs were harvested. Organs were weighed, and samples were digested in aqua regia and Pt concentration was determined using ICP-MS. These studies revealed the majority of the PtNP-DSPE-PEG micelles were sequestered in the liver and spleen (Figure 6). No Pt was detected in the plasma of mice after 24 h, indicating total circulatory clearance of micelles, constant with previous reports of DSPE-PEG micelles having a circulation half-life of 1 to 4 h [27]. Platinum accumulation was observed in all organs tested and ranged from 0.383 ± 0.050 μg/g tissue in the heart, to 1.049 ± 0.332 μg/g in the kidney and 149.044 ± 5.916 μg/g in the spleen. In tumor-bearing mice, the accumulation of Pt was 5.965 ± 4.124 μg/g, which amounted to approximately 3% of the injected dose of Pt.

**Figure 7:**
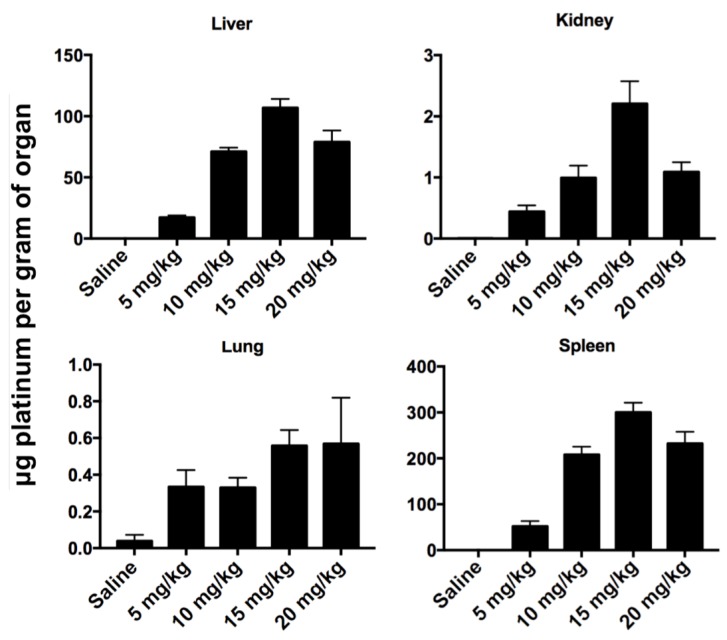
Long-term bioaccumulation study of PtNPs. PtNP:DSPE-PEG micelles were injected into mice with at concentrations of 5–20 mg Pt/kg. After 3 weeks, organs were harvested and quantified for platinum accumulation by inductively-coupled plasma mass spectroscopy (ICP-MS).

Long-term biodistribution studies were performed on animals used for MTD studies. Animals were in injected with 0, 5, 10, 15 and 20 mg Pt/kg body weight (*n* = 3/group), and organs were collected 21 days post-injection. Organs were weighed, and samples were digested for ICP, and are reported as μg Pt per gram organ (Figure 7) or total Pt per organ (Supplementary Materials Figure S2). Platinum concentrations in organs after 3 weeks have a dose response curve up to 15 μg Pt/g, suggesting this reached maximal accumulation of Pt. We analyzed the fraction of injected Pt that is retained after 24 h and 21 days, in mice injected with 10 mg Pt/kg body weight. Table 1 summarizes the percentage of accumulated Pt (total mass Pt per organ divided by injected mass of Pt). No Pt was detected in plasma 24 h after injection indicating that PtNPs had been either excreted from the system or deposited in organs or tissues. Organ accumulation data shows a high deposition of Pt in the liver and spleen after 24-h exposure (20.26% and 6.56%, respectively) and an increase after 3 weeks (40.83% and 9.46%, respectively). This doubling of Pt in the liver would indicate that the micelles are deposited or accumulated in other organs or tissues, and then transported to the liver over the course of weeks, where they are unable to be metabolically processed for excretion or removal. The increase in total Pt in the spleen, a portion of the lymphatic system, suggests that some particles are scavenged though the lymph system and trafficked to the spleen. Kidney accumulation did not change over the course of 3 weeks (0.08%) and lung accumulation decreased from 24-h to 3-week time points (0.10% to 0.03%,) consistent with removal and clearance of accumulated PtNP:DSPE-PEG micelles. Tumor-bearing mice, analyzed 24 h post-injection, show lower accumulation amounts relative to non-tumored mice. The tumors accumulated approximately 3% of the injected Pt (although a high standard deviation is noted), which may account for the lower distributions in the remaining organs.

## 4. Discussion

To move this platform and similar formulations toward translational applications, we must improve our understanding of the induced toxicity, biocompatibility, and fate of nanoparticles. Investigating a novel material construct, such as Pt, as a nano-formulation in biological studies requires a variety of approaches. However, the initial steps in validating its potential in a clinical application should focus on safety and distribution. There are many studies that investigated the effects of PtNP in vitro, but their behavior in vivo after intravenous administration has been limited [3,28,29,30]. To that end, the behavior of Pt-core micelles was explored in vitro and in vivo to determine their feasibility for biomedical applications.

We have developed a method for producing highly uniform PtNP:DSPE-PEG micelles by injecting hydrophobic nanoparticles and lipid-polymers in THF into an aqueous solution. Interestingly, the concentration of the PtNP in the THF assembly mixture appears to be an important factor for the assembled micelle size. The weight-to-weight ratio of PtNPs to DSPE-PEG is a determinant of the final population distribution but appears to be a less important factor than nanoparticle concentration, contrary to our expectation. This suggests that the hydrophobic PtNPs self-assemble during phase transition from THF in the aqueous solution, and lower concentrations of PtNPs produce smaller aggregates; DSPE-PEG then associates with the hydrophobic PtNPs emulsion after assembly but does not significantly contribute to micellar assembly size. We hypothesize that THF acts as short-term surfactant around the hydrophobic PtNP emulsions until the DSPE-PEG stabilizes hydrophobic particles. In the absences of DSPE-PEG, PtNPs injected into an aqueous solution aggregate and precipitate out of solution over the course of hours. This would suggest that micelles have different degrees of DSPE-PEG coating, and the DSPE-PEG associates with the aggregates after assembly and is not the driving force of PtNP aggregation. This potentially alters the amount of surface exposed PEG on the micelles from a “brush” or “mushroom” packing. It should also be noted that the polydispersity index (PDI) was commonly below 0.1 in assembled micelles. No sample generated had a PDI above 0.3, implying sample monodispersity of this micelle assembly method. To move forward into biocompatibility and distribution studies, we chose a micellar assembly that had a less than 100 nm diameter and a high DSPE-PEG coating density. This size is generally accepted to have a good circulation profile, intratumor accumulation rates, and the excess DSPE-PEG coating should efficiently protect the micelle surface from immune response [31].

We began our biological assessment by assaying cytotoxicity in three common cell types, fibroblasts, hepatocytes, and tumor cells. Here the most significant trend observed was detectable cellular toxicity after 24-h exposure in the three cell lines examined. The nanoparticles were well tolerated in all three cell lines for the 1- and 4-h exposures, except for the two highest concentrations in NIH/3T3s showing slight drop after 4 h. Previous PtNP cellular toxicity studies have reported conflicting results stemming from the different synthetic strategies and surface chemistries. Notably, PtNPs with a pristine surface showed little to no adverse cellular effects [19]. A previous report of poly(vinyl pyrrolidone) coated PtNPs showed minimal toxicity effects in untargeted PtNPs compared to a dose-dependent toxicity when a folic acid targeting moiety was incorporated [32]. Similar biocompatibility results have been reported in cancer cell lines using Pt-glutathione nanoclusters [33]. Other studies show varying toxicity at doses approaching the mg/mL range [34,35]. In some cases, toxicity could be attributed to factors such as Pt ion leaching or exotic surface conjugations [36]—which is not expected in formulations using pure, inert Pt cores. Our observations that hydrophobic PtNPs encapsulated in DSPE-PEG show limited cellular toxicity and decreased viability after 24 h is likely due to significant flocculation of the nanoparticles directly onto the cell surface, which is an artifact of 2D tissue culture. This hypothesis is likely confirmed by the comparison of the HepG2 in vitro versus the in vivo liver histology data showing no adverse effects, despite significant uptake and exposure in these tissues at timepoints well beyond 24 h.

As new syntheses of nanomaterials are developed, toxicity and accumulation studies are integral for translational research and application. Based on our in vitro toxicity results using multiple cells lines, we began MTD studies of the PtNP:DSPE-PEG in mice. Micelles were well tolerated in mice, and no weight loss was observed at concentrations up to 20 mg/kg (maximum concentration tested). In comparison to other nanoparticles evaluated in the literature, these PtNP are better tolerated than AgNP, which showed adverse effects at 6 mg/kg [37]. After 21 days, mice were sacrificed, and blood plasma was analyzed for toxicity markers. All results were normal suggesting minimal organ stress or toxicity. The plasma chemistry and histology bolstered the conclusion that the particles had minimal toxicity in vivo. These results are consistent with the previous studies suggesting noble metal particles were unlikely to decompose under biological conditions. A study by Yamagishi et al. showed that nanometer-sized Pt did not induce nephrotoxicity [29], and a similar study from the same group looked at liver toxicity, and revealed similar biocompatibility [30]. However, as noted in the previous section, further investigation of splenic clearance is warranted based on our histological findings.

Biodistribution studies were performed by injecting 10 mg Pt/kg PtNP:DSPE-PEG micelles, and analyzing concentrations of Pt in organs 24 h after administration. We also performed these studies on tumor-bearing mice in anticipation of further efficacy studies. Accumulation of PtNPs in the liver and spleen was expected, as these organs have salient immune functions. Approximately 27% of injected Pt was detected in these two organs after 24 h. Tumor-bearing mice accumulated approximately 3% of the injected Pt in the tumor site, and slightly lower amounts in the liver and spleen relative to naive mice. The passive tumor accumulation showed promise for potential targeting strategies, though intracellular distribution and fate remains an application-dependent barrier. In addition, we analyzed Pt distribution in organs from mice used for MTD studies; these mice received PtNP:DSPE-PEG micelles (5–20 mg Pt/kg) and were analyzed 21 days after injection. In mice that received 10 mg Pt/kg, we observed that the liver and spleen accounted for 50% of the injected Pt. Plasma toxicity markers, including albumin, bilirubin and ALT levels, were all normal, despite high accumulation of platinum in the liver and spleen. We expect that PtNPs accumulate but do not chemically interfere with or affect normal organ functions. In this system, the small PtNPs have an oleylamine shell around each individual crystallite, which we expect prevents aqueous interaction between biological tissues and Pt surfaces.

High nanomaterial accumulation of administered nanomaterials warrants further studies to evaluate how long these particles are retained, and how much material accumulation can be tolerated before markers of toxicity are detectable. As nanomaterial deposition in the liver and spleen may be cumulative if there is no elimination route, we expect this study to be particularly interesting for biomedical applications where multiple administrations are necessary, such as diagnostic applications. Water soluble nanomaterials composed of other elements, including gold and iron, may be more susceptible to metabolic decomposition and elimination, while small inert and highly hydrophobic nanomaterials may be more susceptible to high levels of accumulation. We anticipate exploring these factors in the context of bio-elimination studies in the future, as this is a well know translational impediment for applications of inorganic nanomaterials in medicine.

## 5. Conclusions

Recently, there have been substantial advances in our understanding of the cellular and physiological biology of cancer development and progression, but there remains a need to translate this basic cancer research into effective clinical therapies [38,39]. Nanotechnology opens the door to a wealth of new tools that can be utilized for the development of novel cancer treatments and diagnostics. Nano and molecular therapies that are capable of accumulating at the desired target, while evading the immune system, and minimal off-target effects, should enhance efficacy and reduce side effects for many known cancer therapies. Nanomaterials are particularly well suited for this application, as their size is small enough to remain in circulation for long periods of time (i.e., hours), but large enough to carry substantial cargo. The biological compatibility and biodistribution in vivo of each proposed therapeutic nanomaterial should to be thoroughly tested in order to understand potential biological impact. These results represent an integral step in providing essential information for understanding use of nanomaterials in translational applications.

## Figures and Tables

**Table 1:** Organ distribution of total injected platinum.

Organ	24 h	3 Week	24 h (Tumored Mice)
Liver	20.26% ± 1.347	40.83% ± 2.879	17.48% ± 6.787
Spleen	6.56% ± 0.325	9.46% ± 2.120	5.44% ± 0.534
Kidney	0.08% ± 0.020	0.08% ± 0.026	0.04% ± 0.012
Lung	0.10% ± 0.047	0.03% ± 0.004	0.08% ± 0.041
Tumor	−	−	2.96% ± 1.985

## Supplementary Materials

The following are available online at http://www.mdpi.com/2079-4991/8/6/410/s1, Figure S1: Dynamic light scattering analysis of nanoparticles. Average population size is a function of both the concentration of PtNP in the assembly solution and the weight ratio of PtNPs:DSPE-PEG., Figure S2: Long-term bioaccumulation study of PtNPs. Total platinum per organ after PtNP:DSPE-PEG micelles were administered with at platinum concentrations of 5-20 mg/kg. After 3 weeks organs were harvested and quantified for platinum accumulation by ICPMS.

## References

[1] Arvizo R.R., Bhattacharyya S., Kudgus R.A., Giri K., Bhattacharya R., Mukherjee P. (2012). Intrinsic therapeutic applications of noble metal nanoparticles: Past, present and future. Chem. Soc. Rev..

[2] Kim J.S., Kuk E., Yu K.N., Kim J.H., Park S.J., Lee H.J., Kim S.H., Park Y.K., Park Y.H., Hwang C.Y. (2007). Antimicrobial effects of silver nanoparticles. Nanomedicine.

[3] Yamada M., Foote M., Prow T.W. (2015). Therapeutic gold, silver, and platinum nanoparticles. Wiley Interdiscip. Rev. Nanomed. Nanobiotechnol..

[4] Saha K., Agasti S.S., Kim C., Li X., Rotello V.M. (2012). Gold nanoparticles in chemical and biological sensing. Chem. Rev..

[5] Thakor A.S., Jokerst J., Zavaleta C., Massoud T.F., Gambhir S.S. (2011). Gold nanoparticles: A revival in precious metal administration to patients. Nano Lett..

[6] Brook M.A. (2006). Platinum in silicone breast implants. Biomaterials.

[7] Ghosh P., Han G., De M., Kim C.K., Rotello V.M. (2008). Gold nanoparticles in delivery applications. Adv. Drug Deliv. Rev..

[8] Asharani P.V., Lianwu Y., Gong Z., Valiyaveettil S. (2011). Comparison of the toxicity of silver, gold and platinum nanoparticles in developing zebrafish embryos. Nanotoxicology.

[9] Pedone D., Moglianetti M., De Luca E., Bardi G., Pompa P.P. (2017). Platinum nanoparticles in nanobiomedicine. Chem. Soc. Rev..

[10] Manikandan M., Hasan N., Wu H.F. (2013). Platinum nanoparticles for the photothermal treatment of Neuro 2A cancer cells. Biomaterials.

[11] Porcel E., Liehn S., Remita H., Usami N., Kobayashi K., Furusawa Y., Le Sech C., Lacombe S. (2010). Platinum nanoparticles: A promising material for future cancer therapy?. Nanotechnology.

[12] Wang Z., Chen L., Huang C., Huang Y., Jia N. (2017). Albumin-mediated platinum nanocrystals for in vivo enhanced computed tomography imaging. J. Mater. Chem. B.

[13] Moglianetti M., De Luca E., Pedone D., Marotta R., Catelani T., Sartori B., Amenitsch H., Retta S.F., Pompa P.P. (2016). Platinum nanozymes recover cellular ROS homeostasis in an oxidative stress-mediated disease model. Nanoscale.

[14] Hosaka H., Haruki R., Yamada K., Bottcher C., Komatsu T. (2014). Hemoglobin-albumin cluster incorporating a Pt nanoparticle: Artificial O_2_ carrier with antioxidant activities. PLoS ONE.

[15] Shibuya S., Ozawa Y., Watanabe K., Izuo N., Toda T., Yokote K., Shimizu T. (2014). Palladium and platinum nanoparticles attenuate aging-like skin atrophy via antioxidant activity in mice. PLoS ONE.

[16] Hamasaki T., Kashiwagi T., Imada T., Nakamichi N., Aramaki S., Toh K., Morisawa S., Shimakoshi H., Hisaeda Y., Shirahata S. (2008). Kinetic analysis of superoxide anion radical-scavenging and hydroxyl radical-scavenging activities of platinum nanoparticles. Langmuir.

[17] Khlebtsov N., Dykman L. (2011). Biodistribution and toxicity of engineered gold nanoparticles: A review of in vitro and in vivo studies. Chem. Soc. Rev..

[18] Alkilany A.M., Murphy C.J. (2010). Toxicity and cellular uptake of gold nanoparticles: What we have learned so far?. J. Nanopart. Res..

[19] Horie M., Kato H., Endoh S., Fujita K., Nishio K., Komaba L.K., Fukui H., Nakamura A., Miyauchi A., Nakazato T. (2011). Evaluation of cellular influences of platinum nanoparticles by stable medium dispersion. Metallomics.

[20] XTF E., Zhang Y., Zou J.-J., Wang L., Zhang X. (2014). Oleylamine-Protected Metal (Pt, Pd) Nanoparticles for Pseudohomogeneous Catalytic Cracking of JP-10 Jet Fuel. Ind. Eng. Chem. Res..

[21] Zhang X.D., Wu D., Shen X., Liu P.X., Fan F.Y., Fan S.J. (2012). In vivo renal clearance, biodistribution, toxicity of gold nanoclusters. Biomaterials.

[22] Zarschler K., Rocks L., Licciardello N., Boselli L., Polo E., Garcia K.P., De Cola L., Stephan H., Dawson K.A. (2016). Ultrasmall inorganic nanoparticles: State-of-the-art and perspectives for biomedical applications. Nanomedicine.

[23] Gref R., Luck M., Quellec P., Marchand M., Dellacherie E., Harnisch S., Blunk T., Muller R.H. (2000). ‘Stealth’ corona-core nanoparticles surface modified by polyethylene glycol (PEG): Influences of the corona (PEG chain length and surface density) and of the core composition on phagocytic uptake and plasma protein adsorption. Colloids Surf. B Biointerfaces.

[24] Jokerst J.V., Lobovkina T., Zare R.N., Gambhir S.S. (2011). Nanoparticle PEGylation for imaging and therapy. Nanomedicine (Lond.).

[25] Cafaggi S., Russo E., Stefani R., Leardi R., Caviglioli G., Parodi B., Bignardi G., De Totero D., Aiello C., Viale M. (2007). Preparation and evaluation of nanoparticles made of chitosan or N-trimethyl chitosan and a cisplatin-alginate complex. J. Control. Release.

[26] River C. BALB/C Mouse Hematology. https://www.criver.com/sites/default/files/resources/BALBcMouseClinicalPathologyData.pdf..

[27] Che J., Okeke C.I., Hu Z.B., Xu J. (2015). DSPE-PEG: A distinctive component in drug delivery system. Curr. Pharm. Des..

[28] Rai M., Ingle A.P., Gupta I., Brandelli A. (2015). Bioactivity of noble metal nanoparticles decorated with biopolymers and their application in drug delivery. Int. J. Pharm..

[29] Yamagishi Y., Watari A., Hayata Y., Li X., Kondoh M., Yoshioka Y., Tsutsumi Y., Yagi K. (2013). Acute and chronic nephrotoxicity of platinum nanoparticles in mice. Nanoscale Res. Lett..

[30] Yamagishi Y., Watari A., Hayata Y., Li X., Kondoh M., Tsutsumi Y., Yagi K. (2013). Hepatotoxicity of sub-nanosized platinum particles in mice. Pharmazie.

[31] Blanco E., Shen H., Ferrari M. (2015). Principles of nanoparticle design for overcoming biological barriers to drug delivery. Nat. Biotechnol..

[32] Teow Y., Valiyaveettil S. (2010). Active targeting of cancer cells using folic acid-conjugated platinum nanoparticles. Nanoscale.

[33] Chen D., Gao S., Ge W., Li Q., Jiang H., Wang X. (2014). One-step rapid synthesis of fluorescent platinum nanoclusters for cellular imaging and photothermal treatment. RSC Adv..

[34] Mohammadi H., Abedi A., Akbarzadeh A., Mokhtari M.J., Shahmabadi H.E., Mehrabi M.R., Javadian S., Chiani M. (2013). Evaluation of synthesized platinum nanoparticles on the MCF-7 and HepG-2 cancer cell lines. Int. Nano Lett..

[35] Hashimoto M., Yamaguchi S., Sasaki J., Kawai K., Kawakami H., Iwasaki Y., Imazato S. (2016). Inhibition of matrix metalloproteinases and toxicity of gold and platinum nanoparticles in L929 fibroblast cells. Eur. J. Oral Sci..

[36] Alshatwi A.A., Athinarayanan J., Vaiyapuri Subbarayan P. (2015). Green synthesis of platinum nanoparticles that induce cell death and G2/M-phase cell cycle arrest in human cervical cancer cells. J. Mater. Sci. Mater. Med..

[37] De Jong W.H., Van Der Ven L.T., Sleijffers A., Park M.V., Jansen E.H., Van Loveren H., Vandebriel R.J. (2013). Systemic and immunotoxicity of silver nanoparticles in an intravenous 28 days repeated dose toxicity study in rats. Biomaterials.

[38] Anselmo A.C., Mitragotri S. (2016). Nanoparticles in the clinic. Bioeng. Transl. Med..

[39] Anselmo A.C., Mitragotri S. (2015). A Review of Clinical Translation of Inorganic Nanoparticles. AAPS J..

