# Supplementary material for: Biodistribution and Toxicity of Micellar Platinum Nanoparticles in Mice via Intravenous Administration
Source: Nanomaterials (Basel). 2018 Jun 7;8(6):410. doi: 10.3390/nano8060410 (PMC6027383; doi:10.3390/nano8060410)
Supplement: Supplementary file 1 [file nanomaterials-08-00410-s001.pdf]

Supplemental Material

Biodistribution and toxicity of micellar platinum nanoparticles in mice via intravenous administration

Anna Brown[a,1], Marc Kai [a,1], Allison DuRoss [a], Gaurav Sahay [a,b], and Conroy Sun [a,c,*]

[a] Department of Pharmaceutical Sciences, College of Pharmacy, Oregon State University, 2730 SW Moody Ave, Portland, OR 97201, USA
[b] Department of Biomedical Engineering, School of Medicine, Oregon Health & Science University, 2730 SW Moody Ave, Portland, OR 97201, USA
[c] Department of Radiation Medicine, School of Medicine, Oregon Health & Science University, 3181 S.W. Sam Jackson Park Road, Portland, OR 97239, USA

[1] These authors contributed equally to this work.

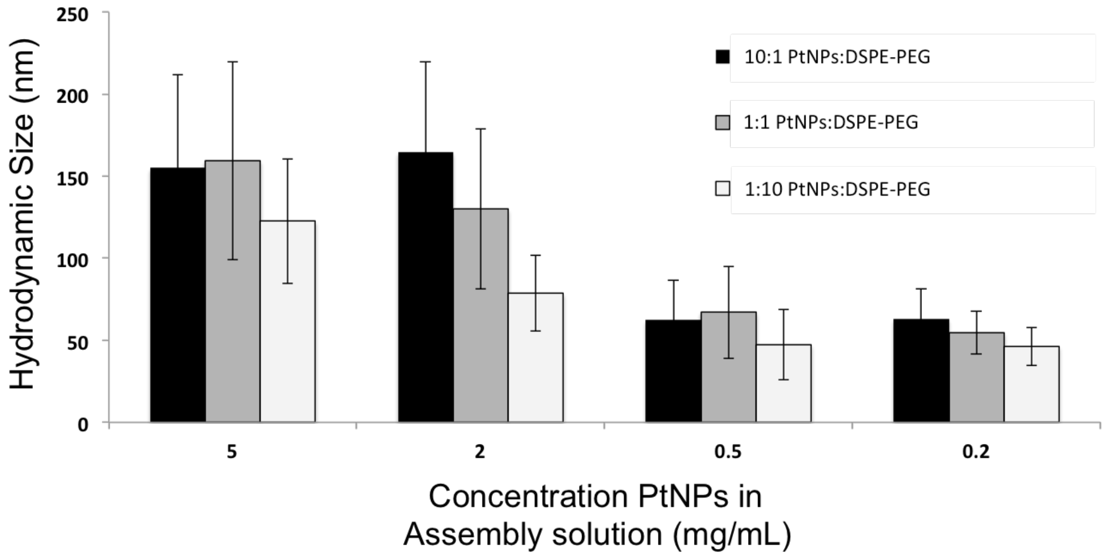

Supplemental Figure 1. Dynamic light scattering analysis of nanoparticles. Average population size is a function of both the concentration of PtNP in the assembly solution and the weight ratio of PtNPs:DSPE-PEG.

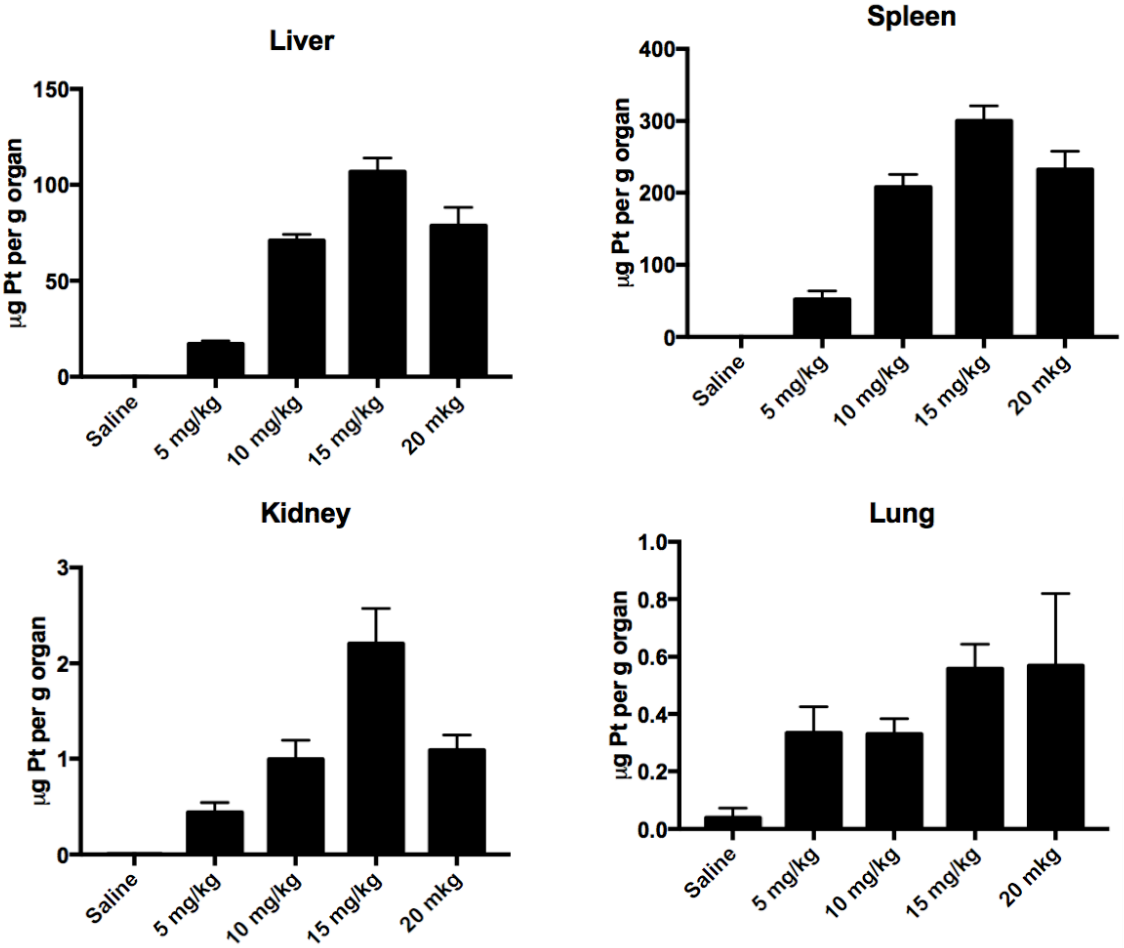

Supplemental Figure 2. Long-term bioaccumulation study of PtNPs. Total platinum per organ after PtNP:DSPE-PEG micelles were administered with at platinum concentrations of 5-20 mg/kg. After 3 weeks organs were harvested and quantified for platinum accumulation by ICP-MS.
